# Conservative treatment for well-differentiated endometrial cancer: when and why it should be considered in young women

**DOI:** 10.3332/ecancer.2019.892

**Published:** 2019-01-16

**Authors:** Michele Peiretti, Francesca Congiu, Enzo Ricciardi, Paolo Maniglio, Valerio Mais, Stefano Angioni

**Affiliations:** 1 Department of Surgical Sciences, Division of Gynecology and Obstetrics, University of Cagliari, 09124 Cagliari, Italy; 2 Department of Gynäkologie and Gynäkologische Onkologie, Kliniken Essen-Mitte, 45136 Essen, Germany; 3 Department of Scienze Medico-Chirurgiche e di Medicina Traslazionale, Sapienza University of Rome, 00185 Rome, Italy

**Keywords:** endometrial cancer, fertility-sparing treatment, hysteroscopic resection, conservative treatment

## Abstract

The aim of this review was to update current knowledge on the conservative treatment of endometrial cancer (EC) based on a literature review. A web-based search in the MEDLINE database was carried out on EC management and treatment. All relevant information has been collected and analysed. Case series were mainly found in the literature search. Conservative treatments were offered to young patients with stage I low-grade endometrioid carcinomas of the endometrium. Different options included high/low dose progestin treatments, hysteroscopic resection of the disease, a levonorgestrel intrauterine device or a combination of various strategies. The overall complete response rate was near 76.5% with a recurrence rate of up to 33.8%. Pregnancy outcomes reached rates of 64.8% for live births. The current clinical outcomes show that conservative treatment aimed at preserving fertility is feasible for stage I endometrial well-differentiated adenocarcinomas in motivated patients under close monitoring.

## Introduction

Endometrial cancer (EC) is the most common gynaecologic malignancy in the United States. It accounts for 3.6% of all cancers (incidence rate of 26.1/100,000 women) and it generally affects postmenopausal women, with a median age at diagnosis of 62 years [1]. EC tends to be diagnosed at an early stage in most women and is usually associated with a good prognosis. Transvaginal ultrasound (US) and diagnostic hysteroscopy are the main diagnostic procedures in the diagnosis. Hysteroscopy provides direct visualisation of the endometrial cavity, thereby allowing a targeted biopsy or excision of the lesions identified during the procedure [2]. Miniaturised devices and the development of an office approach allow an accurate and reliable diagnosis in the majority of women with abnormal uterine bleeding or with abnormal uterine findings at USs [3]. Abnormal uterine bleeding is the most common symptom and a preoperative diagnosis should be mandatory to avoid unexpected malignancy and consequently, inappropriate surgical treatment and possible spreading of the disease [4]. Standard treatment of early-stage EC includes hysterectomy without vaginal cuff, bilateral salpingo-oophorectomy with sentinel lymph node mapping or systematic pelvic and para-aortic lymph node dissection [10, 11]. In those patients older than 45 years with G2-G3 and myometrial invasion, bilateral salpingo-oophorectomy is recommended to avoid the risk of stimulating an undetected metastatic disease [4].

Young patients generally have a history of oligomenorrhoea, chronic anovulation and infertility, obesity, polycystic ovary syndrome (PCOS) and diseases related to the exposure of oestrogens and androstenedione [5–7]. Moreover, they tend to have a more favourable prognosis considering the frequent low grading and no evidence of myometrial invasion [6, 8, 9]. In fact, when the disease is well differentiated and confined to the uterus, the 5-year survival rate is 96% [1].

The endometrioid type is the most prevalent, accounting for 90% of all cases. High-grade tumours, such as the clear cell and undifferentiated types, are present in the remaining cases and these are considered characteristic of patients of an advanced age [4].

Young patients who are willing to preserve fertility may prefer a conservative treatment and should be carefully evaluated with appropriate consultation. A myometrial invasion and the grading of the neoplasm should be identified, these being the main prognostic factors and indications for a possible conservative treatment [4]. Despite the fact that these two parameters are good indicators of the spread of the disease, patients with grade 1 disease have about 3% and 2% of pelvic and para-aortic dissemination, respectively [13]. In cases of grade 1 lesions that are confined to the endometrium, the rate of nodal metastasis is negligible. Therefore, the exclusion of any myometrial involvement in grade 1 lesions should be regarded as an indication for conservative treatment [14].

Several studies have tried to evaluate a more specific and sensitive test in evaluating the involvement of the myometrium. Hardesty et al [15] reported a comparison between magnetic resonance imaging (MRI) and spiral computed tomography (CT) scans, showing that MRI is more sensitive and specific in the detection of myometrial invasion and cervical involvement. Comparisons between MRI and transvaginal US showed that both these techniques produce similar results [16, 17]. Saez *et al* [18] reported a higher diagnostic accuracy of the MRI with gadolinium. This same group also confirmed findings that emphasise the better diagnostic results obtained through the MRI with contrast agents [19]. Literature data show MRI sensitivity and specificity of 83% and 82%, respectively, so it can be considered an effective but still not a perfect diagnostic tool [20].

Therefore, all patients opting for a conservative treatment have to be adequately informed of the possibility of false negatives and their relative consequences. For those patients who are candidates for conservative treatment, a laparoscopy with peritoneal cytology, lymph node biopsy and an evaluation of the adnexa should be offered [12].

The following criteria must be met for appropriate screening of young patients wanting to preserve fertility:

- Well-differentiated carcinoma
- Myometrial invasions absent on the MRI, CT scan and pelvic US (trans-abdominal–trans-vaginal)
- No pelvic and para-aortic lymph node involvement
- No ovarian cancer
- No contraindications to medical treatments
- Patients who strongly wish to preserve fertility

In addition, the patient has to agree to attend a precise follow-up after the treatment.

The aim of the present review consists of an evaluation of the various medical strategies for well-differentiated early-stage EC that a gynaecologic oncologist should offer to these women. We particularly focused on contemporary clinical trials and the evidence surrounding conservative management.

## Materials and methods

An electronic literature search via the MEDLINE database was performed to identify published English-language articles addressing the conservative treatment of women with a histologic diagnosis of grade 1 adenocarcinoma of the endometrium.

We selected reports published between January 2004 and December 2017 using the PubMed database. Keywords and terms searched included: conservative treatment, endometrial cancer, progestin therapy and fertility. The search was further narrowed by limiting articles cited to only those reporting on humans.

**Figure 1.**
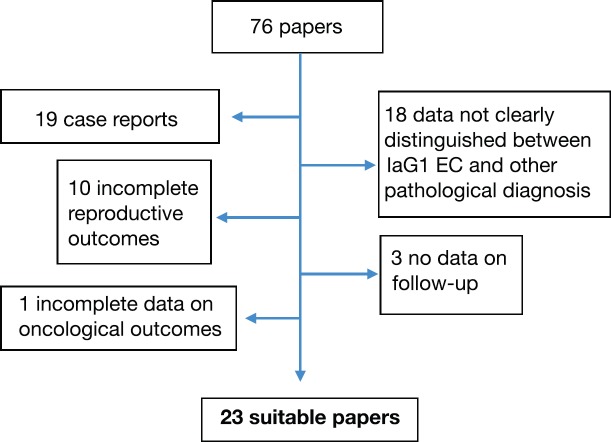
Selection of suitable papers for our review.

We excluded studies that described hormonal therapy in the following settings: high-risk (grade 2 or 3) endometrial adenocarcinoma, advanced-stage EC and non-endometrioid type. We also excluded case reports or papers in which EC data were not clearly distinguished from atypical hyperplasia (AH) ones or in which considered patients included also postmenopausal women. Furthermore, papers with incomplete results on oncological or pregnancy outcomes were excluded. The results of selected papers were carefully evaluated to establish their relevance. (Figure 1).

From each relevant study, we analysed demographic data: patient’s age, body mass index (BMI), parity, comorbidities, histology, different types of hormonal agents, routes of administration and method and timing of intervals for endometrial re-evaluations. Information regarding oncologic and fertility outcomes was recorded, including response rates. A response of the grade 1 adenocarcinoma of the endometrium was defined as ‘complete regression’ when the final specimen showed an atrophic, proliferative or secretory endometrium. When no change was detected in histological specimens, we defined it ‘stable disease’, when pathology showed regression of lesions from Cancer to Hyperplasia, a ‘partial remission’ was defined. ‘Progressive disease’ was diagnosed when follow-up tests demonstrated a higher grade of disease or myometrial invasion, while ‘Relapse’ was defined as positivity of pathological analysis after one or more negative specimens.

Data regarding reproductive outcomes were also collected, including the number of patients who became pregnant and the number of live births, both from patients who conceived spontaneously and with assisted reproductive technologies (ART); however, this information was not reported in all studies.

We analysed results from prospective/retrospective studies published to date, focusing on all controversies surrounding fertility-sparing treatment for well-differentiated EC.

## Results

We identified 23 studies, including prospective/retrospective series. From these studies, we identified 299 patients with a diagnosis of well-differentiated endometrial adenocarcinoma who were treated with various hormonal scheduled therapies, combined, in some studies, with hysteroscopic resection of the tumour.

The median age for the overall cohort was 31, 45 years (range: 18–47 years). Median BMI was 24 (range 17.4–77.6). Data regarding parity were available only for 184 patients. Of these, 148 patients (79%) were nulliparous. Seventeen studies reported associated conditions, as PCOS, menstrual irregularities, diabetes mellitus, hypertension, familiar history positive for Lynch syndrome or other tumours. (Demographic data and treatment features are showed in Table 1.)

Hysteroscopic resection of neoplastic lesions preceded medical treatment in four studies (76 patients, 25% of total).

The most commonly used drugs were megestrol acetate, medroxyprogesterone acetate (MPA), levonorgestrel (LNG) intrauterine device (IUD), aromatase inhibitors (letrozole, anastrozole), analogue of gonadotropin-releasing hormone (GnRH) (as leuprolide), combined oral contraceptives, lynestrenol, tamoxifen, norethisterone acetate and hydroxyprogesterone caproate. In most studies, more than one drug was used, so it was not possible to specify how many patients took every drug.

After a median follow-up of 39 months (range: 1.5–412 months), the complete regression rate was 76.5% (228 patients) with a relapse rate of 33.5% (77 patients) between patients who had a complete response (CR).

Oncological outcomes are shown in Table 2. Sixty-five patients (21.7%) did not respond to therapy. Of these patients, 33 (50.8%) showed stable disease (SD) and 32 (49.2%) showed progressive disease (PD). Seven patients (2.3%) experienced partial remission (PR).

Follow up consisted in dilatation and curettage (D&C) (69.6% of studies) or any other endometrial sampling (as hysteroscopic biopsy or Pipelle) in 100% of studies, in 11 studies in transvaginal US (47.8% of studies), MRI (26% of studies), at a median interval between evaluations of 3 months.

Data regarding surgical management are the following: 136 women (45.6%) had a hysterectomy. Only four studies reported the interval of time between diagnosis and hysterectomy. This interval ranged from 6 to 84 months, with a median of 12 months. Finally, data on histology were reported for 132 women. Of these, 33 had negativity for any malignancy, 59 revealed IAG1 EC, 9 showed PR to AH and 22 showed progression (because of increased grading or staging). In particular, nine women had simultaneous ovarian implants of endometrioid adenocarcinoma.

Data regarding adverse effects were collected. Only six studies reported adverse effects, and six specified the absence of any side effect. Among the studies considered, five reported weight gain, three reversible liver dysfunction, one swelling and cutaneous hyperpigmentation and one study reported hot flushes and vaginal dryness (this study contemplated the use of GnRH analogues).

**Figure 2.**
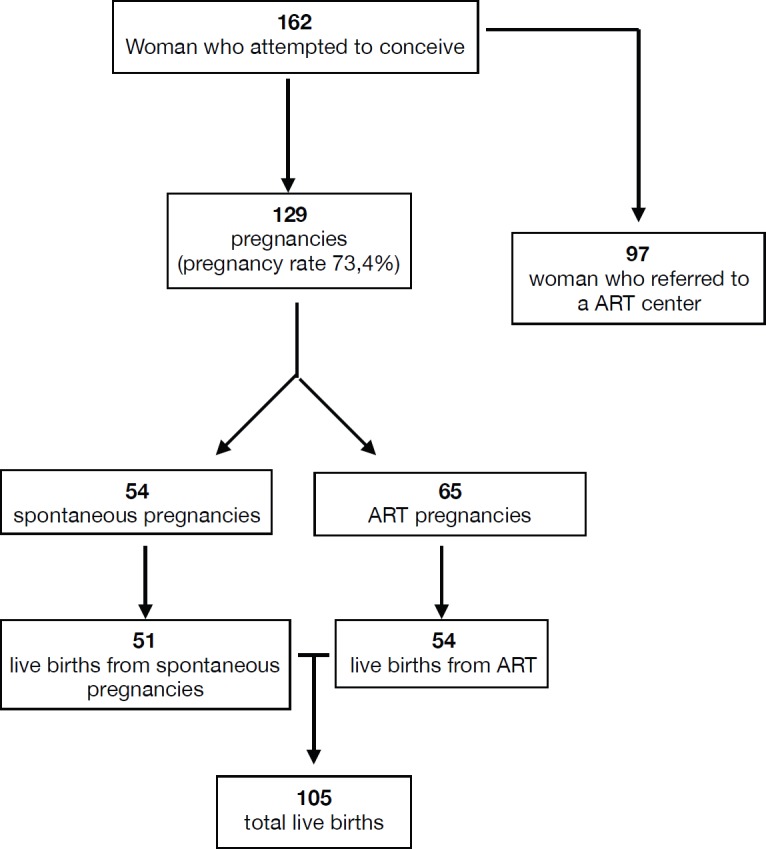
Reproductive outcomes.

Regarding reproductive outcome, data are shown in Figure 2. Studies reported a total of 119 pregnancies (among 162 women who attempted to conceive, pregnancy rate 73.4%), which included women who conceived both spontaneously (54 pregnancies) and via ART (65). In particular, 97 women referred to the ART centre. Of the 119 analysed pregnancies, 105 live births occurred (54 from ART).

## Discussion

Given the hormonal dependency of EC, several groups have mainly adopted medical treatments with high doses of progestins, while others utilised gonadotropin-releasing hormone agonists (GnRHa) or antiandrogens [21–23]. It has been well established that progestins, besides many other systemic effects, may cause endometrial atrophy and are widely used in the treatment of many gynaecological benign conditions [24]. Considering the hormonal dependency of the majority of ECs, hormonal therapy could lead to a tumoural quiescence or even a disappearance with treatment [25]. Specific mechanisms explaining how progestins acting as oestrogen antagonists can cause their effect on endometrial carcinomas could be related to apoptosis pathways [25] and more the recently described Phospatase and Tensin Homolog gene mutation pathways [24].

Progestins have been utilised for years in the treatment of metastatic EC because they act as oestrogen antagonists and inhibit the mitosis of endometrial cells and favour apoptosis [26]. Some of these also have anti-angiogenic effects [27–29]. Several groups, as described below, have utilised MPA 100–800 mg one time per day (QD) for 4–14 months.

Kaku *et al* [21] reported treating 12 patients with EC with MPA 200–400 mg QD. Of the 12 patients, eight had a CR to treatment and one patient had a partial response. The average length of treatment was 3 months. The average follow-up examination occurred at 24 months for responding patients and showed a case of recurrence treated with a second cycle of MPA that responded to therapy. Ferrandina *et al* [30] reported on a 30-year-old patient who was nulliparous with a well-differentiated endometrial adenocarcinoma. This patient was treated from the 15th to the 25th day following the onset of menstruation with dihydrogesterone 20 mg QD. The patient subsequently responded to medical treatment and had a successful pregnancy and delivery. Eight months later, regular follow-up MRI and CT scan showed distant metastases and an exploratory laparotomy revealed parametrial, ovarian, cervical and omental metastases. Kimming *et al* [31] reported a case of a 28-year-old patient who was nulliparous with a well-differentiated adenocarcinoma treated with MPA 200 mg QD for 8 weeks. After medical treatment, the diagnostic hysteroscopy and biopsy revealed pathology regression. The patient then underwent gamete intrafallopian transfer and successfully carried triplets to term and delivered three live foetuses. A year after delivery, the patient did not show any signs of recurrent disease or distant repetitions. Randall and Kurman reported on a study conducted on 12 women with well-differentiated adenocarcinomas who were younger than 40 years of age and treated with high doses of progestins. Nine patients had a CR to treatment after 9 months, however, three patients still had the disease. None of the responding patients showed signs of recurrent or progressing disease after an average follow-up of 40 months [32]. Yamazawa *et al* [8] reported on nine women averaging 36 years of age with endometrial adenocarcinomas and treated with MPA 400 mg QD for 6 months. Of the nine patients, 78% had a CR and 22% a partial one. The overall rate of response was 100% after 6 months of treatment with MPA. Ramirez *et al* [33] published a review comprised of 27 publications on the conservative treatment of 81 patients. Half of the patients were treated with MPA 200–600 mg QD. Although approximately 75% of the patients had a CR to treatment within an average of 12 weeks, the average length of treatment was 6 months. Furthermore, 76% had a recurrence after an average of 20 months and 30% of these patients with recurrence underwent a second cycle of progestins with a response in 80% of the cases. We may conclude that for half of the cases, fertility was preserved, thanks to hormonal therapy with high doses of progestin, and that 25% of the cases that failed to respond to the first therapeutic cycle had complete recovery after the second cycle.

After a detailed analysis of the previous clinical experiences, there is no definitive consensus regarding the optimal progestin regimen and timing, and most studies assessed in this review did not report long follow-up times following hormonal treatments. Progestin therapy has an impact on the endometrial cells as early as 10 weeks after the initiation of treatment. However, most recognise the need for a minimum of 3 months of treatment before assessing a response with endometrial hyperplasia and even longer for EC [34–36]. Thus, some clinicians use this time frame in determining responsiveness to progestin therapy.

An innovative approach to the conservative treatment of endometrial carcinomas requires hysteroscopic resection of EC along with part of the adjacent myometrium and subsequent medical therapy with progestin. Two Italian series described a total of 20 patients treated with a similar hysteroscopic technique in which the authors observed only one recurrence after a median follow-up of 40 and 50.5 months in five patients with six pregnancies. This technique may be considered for those cases with small lesions even though there are possible complications, such as spreading of the disease, intrauterine adhesions and obstetric complications due to myometrial damage [37–39]. In particular, placenta accreta is the most dangerous eventuality in these patients because of the altered vascularity of damaged tissues and higher invasivity of trophoblast in scars [40]. However, it should be considered in selected cases. A recent review of the literature reported a recurrence rate of 11% after a median follow-up period of 40 months (range: 11–82 months) after hysteroscopic resections of endometrial lesions and subsequent hormonal therapy with oral and/or IUD progestins [41].

Another interesting therapeutic option is the use of the levonorgestrel-releasing-IUD (LNG-IUD) plus GnRH. Some case reports have been published with a 100% CR rate [42, 43]. Minig L *et al* [44] treated 34 patients with a combination of LNG-IUD for 1 year followed by 6 months of GnRH treatment in patients younger than 40 years of age. The CR rate was 95%, although the relapse rate among patients with atypical endometrial hyperplasia was 5% compared to 28% among patients diagnosed with EC.

To find the best evidence, we need to wait for the results of the following two trials (both recruiting patients): phase II trial from MD Anderson (registry # NCT0078861) on the use of LNG-IUD for the treatment of endometrial hyperplasia and low-grade endometrial carcinomas, and the feMMe trial, by Obermair *et al* [45] (registry # NCT01686126), which aims to study the efficacy and safety of LNG-IUD + Metformin + weight loss in patients at an early stage of EC. These studies could clarify a specific role for the use of LNG-IUD in this disease.

Data selected for our review show a significant response rate for EC patients treated conservatively (76.25% achieved CR), but there is a significant relapse rate—33.8% of patients who had a complete remission). Thus, it seems fundamental to adequately select patients and carefully counsel them before starting a conservative approach. It is also necessary to plan a comprehensive follow-up to diagnose eventual disease stability (50.8% of non-responders (NR) in our review), progressions (49.2% of NR) or relapses and propose the patient for further medical therapy or standard surgery.

After a complete pathological response, all patients should attempt pregnancy. In those women who do not become pregnant after 3 months, it is advised to carry out first-stage examinations for the evaluation of an infertile couple. If the woman has a history of infertility or chronic anovulation and has no signs of disease, ovulation induction may be considered. Despite the exposure to high levels of oestrogens, a higher risk of recurrence has not been shown; in fact, the utilisation of MPA may reduce the waiting time of those wanting a pregnancy [46–49].

From our international literature review, we found a wide range of pregnancy successes (overall pregnancy rate 73.4%) among young women with EC after conservative treatment. The tumours were well differentiated in all of the selected studies, and the pregnancies were obtained through ART in 51.4% of the cases.

All patients should consider demolitive surgery as soon as possible after achieving pregnancy or after the failure of several ART procedures.

In our set of data, hysterectomy with pathological evaluation was reported for 132 (44%) patients. The surgical procedure was performed after a medium period of 30 months after diagnosis, reflecting the interval necessary for therapy and ART to be accomplished. Between all the patients who underwent surgical procedure, only 33 patients (25%) showed no evidence of disease at pathological evaluation, while 22 (16.7%) showed a progression of the disease, thus pointing out the importance of surgery in all patients diagnosed with EC, even if conservative therapy was effective in a short period.

There are some limitations to this review. First, the main source was a group of retrospective studies, there may have been inherent reporting and observational biases and a lack of long-term follow-up data for many studies. Additionally, details of treatments and surgical pathologies were often not available and there was no centralised pathological review.

Some included studies were not of high quality, so we conclude that large prospective or randomised studies comparing the efficacy of hormonal agents and associated reproductive outcomes are warranted.

## Conclusion

Conservative treatment with medical therapy with or without hysteroscopic resection of the lesion should be considered in selected young patients with grade 1 stage I EC who wish to preserve fertility. These patients should undergo comprehensive follow-up after achievement of CR. This is important because, despite high CR rates, it seems fundamental to identify patients who did not respond, who had PD or who relapsed.

Patients should also attempt pregnancy as soon as possible after the CR, spontaneously or via ART, to minimise the risk of relapse.

After the achievement of pregnancy, or after the failure of ART, standard surgery should be offered to patients.

## Funding and conflicts of interest

The authors of the present publication did not receive any research support funding and declare no conflict of interest.

## Figures and Tables

**Table 1.** Demographic data and treatment features.

Demographic data (*n* = 299)		
Median age	31, 45 years	18–47 years
Median BMI	24	17.4–77.6
Nulliparous (data available only for 184 patients)	148	79%
Hysteroscopic resection prior to medical treatment	4 studies, 76 patients	25%
Pharmacological agents **I.** Megestrol Acetate **II.** MPA **III.** LNG-IUD **IV.** Letrozole/Anastrozole **V.** GnRH-a (Leuprolide) **VI.** Combined Oral Contraceptives **VII.** Lynestrenol **VIII.** Tamoxifen (always in association with other drugs) **IX.** Norethisterone acetate **X.** Hydroxyprogesterone caproate	18 studies 14 studies 5 studies 1 study 2 studies 1 study 1 study 2 studies 2 studies 1 study	
Median duration of therapy	6 months	2–49 months

**Table 2.** Oncological outcomes.

Oncological outcomes (*n* = 299)		
CR	228	76.25% of total
NR	65	21.7% of total
SD	33	50.8% of NR
PD	32	49.2% of NR
PR	7	2.3% of total
Relapse	77	33.8% of CR
